# TMSC-m7G: A transformer architecture based on multi-sense-scaled embedding features and convolutional neural network to identify RNA N7-methylguanosine sites

**DOI:** 10.1016/j.csbj.2023.11.052

**Published:** 2023-12-01

**Authors:** Shengli Zhang, Yujie Xu, Yunyun Liang

**Affiliations:** a School of Mathematics and Statistics, Xidian University, Xi’an 710071, PR China; b Key Laboratory of Computational Science and Application of Hainan Province, Haikou 571158, PR China; c School of Science, Xi’an Polytechnic University, Xi’an 710048, PR China

**Keywords:** RNA N7-methylguanosine, Natural language processing, Word embedding, Transformer, Convolutional neural network

## Abstract

RNA N7-methylguanosine (m7G) is a crucial chemical modification of RNA molecules, whose principal duty is to maintain RNA function and protein translation. Studying and predicting RNA N7-methylguanosine sites aid in comprehending the biological function of RNA and the development of new drug therapy regimens. In the present scenario, the efficacy of techniques, specifically deep learning and machine learning, stands out in the prediction of RNA N7-methylguanosine sites, leading to improved accuracy and identification efficiency. In this study, we propose a model leveraging the transformer framework that integrates natural language processing and deep learning to predict m7G sites, called TMSC-m7G. In TMSC-m7G, a combination of multi-sense-scaled token embedding and fixed-position embedding is used to replace traditional word embedding for the extraction of contextual information from sequences. Moreover, a convolutional layer is added in the encoder to make up for the shortage of local information acquisition in transformer. The model's robustness and generalization are validated through 10-fold cross-validation and an independent dataset test. Results demonstrate outstanding performance in comparison to the most advanced models available. Among them, the Accuracy of TMSC-m7G reaches 98.70% and 92.92% on the benchmark dataset and independent dataset, respectively. To facilitate the popularization and use of the model, we have developed an intuitive online prediction tool, which is easily accessible for free at http://39.105.212.81/.

## Graphical Abstract

**Figure:**
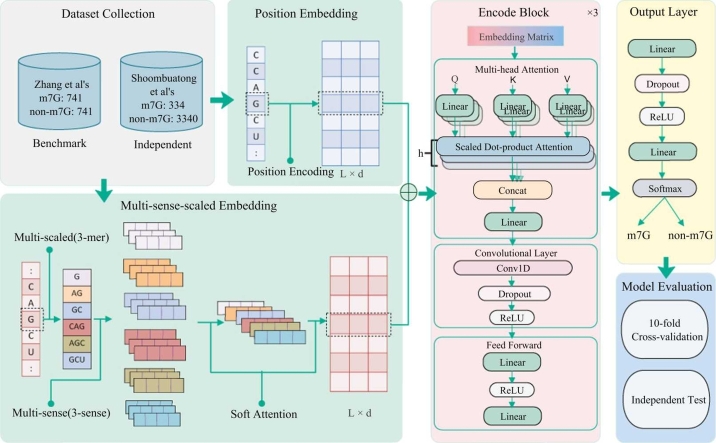

## 1 Introduction

N7-methylguanosine (m7G) is a prevalent post-transcriptional modification in RNA [1], playing a pivotal role in maintaining RNA functionality and protein translation [2], [3]. Positioned at the seventh nitrogen site within the RNA molecule, it is predominantly located upstream of the 5′ cap in RNA sequences [4], [5], [6]. This modification involves methylating the N7 position of the guanosine base. The post-transcriptional mechanism of m7G is orchestrated by methyltransferases [7], which regulate the expression levels of m7G through the catalytic methylation of specific nitrogen sites. This methylation event plays a critical role in modulating RNA functionality, influencing processes such as RNA stability, translation efficiency, and overall gene expression regulation [8], [9], [10], [11], [12], [13], [14]. The precise positioning and modification of m7G contribute significantly to its functional relevance in the intricate network of post-transcriptional RNA modifications. N7-methylguanine is also found inside tRNA and rRNA molecules [15]. Furthermore, several studies have shown the possible implication of RNA m7G modification dysfunction in various diseases, including growth deficiency [16], microcephalic primordial dwarfism, brain malformation, as well as the development of certain autoimmune disorders [17], [18]. Methods for predicting m7G sites in RNA molecules can help scientists better understand the significance of RNA modification in life. Therefore, understanding the distribution and changes of RNA m7G sites is of great significance for further unraveling the link between RNA modification and disease.

With the continuous progress of high-throughput sequencing technology, there has been a growing focus among biologists on developing prediction methods for RNA m7G sites. In the biological experiments for m7G identification, cell lines are predominantly used for experimental research. The primary reason for this choice is that acquiring, processing, and maintaining human tissue samples can be prohibitively expensive and complex. In contrast, cell lines offer a more cost-effective and efficient means to conduct large-scale experimental studies. Some experimental methods for predicting m7G sites have been used, such as AlkAniline-seq by Marchand et al. [19], MeRIP-seq by Zhang et al. [20], and miCLIP-seq by Lionel et al. [21]. However, given the complexity, time consumption, and resource intensiveness of these experimental methods, there's a necessity to devise computational approaches to predict m7G sites efficiently and accurately.

Currently, machine learning and pattern recognition methods are extensively employed for categorizing biological sequences. Work [22] investigates improvements in biosequence pattern-matching algorithms, comparing and analyzing different algorithms for biosequence-based pattern recognition from aspects of complexity, efficiency, and technology. Work [23] introduces a novel rapid biosequence pattern-matching technique. Work [24] optimizes the classification efficiency of DNA sequences using machine learning techniques. Current computational methods can predict m7G sites by using sequence and structural information and applying machine learning and deep learning techniques. Chen et al. [25] used the support vector machine (SVM) to build the first machine learning predictor, called iRNA-m7G, which integrated three sequence-based features from different perspectives for training. Subsequently, Song et al. [26] proposed m7GFinder, taking into account characteristics derived from both sequences and genomes and also using SVM for prediction. Bi et al. [27] introduced XG-m7G, employing six different approaches for sequence representation in conjunction with the XGBoost algorithm for the identification of m7G sites. Dai et al. [7] developed m7G-IFL, which introduces a novel iterative approach for encoding RNA sequences, enabling automatic learning of probability distribution information across various sequence models and enhancing the feature representation capability. Shoombuatong et al. [28] proposed THRONE, constructed an independent dataset for testing and used a variety of machine learning algorithm integration to predict m7G sites, achieving excellent results. Despite the endless emergence of m7G site predictors, there are still some limitations in current studies: (1) Most predictors use fixed encoding to represent features, which is not conducive to the capture of high-potential information. (2) Traditional machine learning methods are still the mainstream, while advanced deep learning methods have not been adopted yet. In recent years, satisfactory outcomes have been observed in multiple studies that have explored the capability of natural language processing (NLP) technology to transform biological sequences, encompassing DNA and protein sequences, into vectorized feature representations [29], [30], [31], [32], [33], [34].

**Fig. 1:**
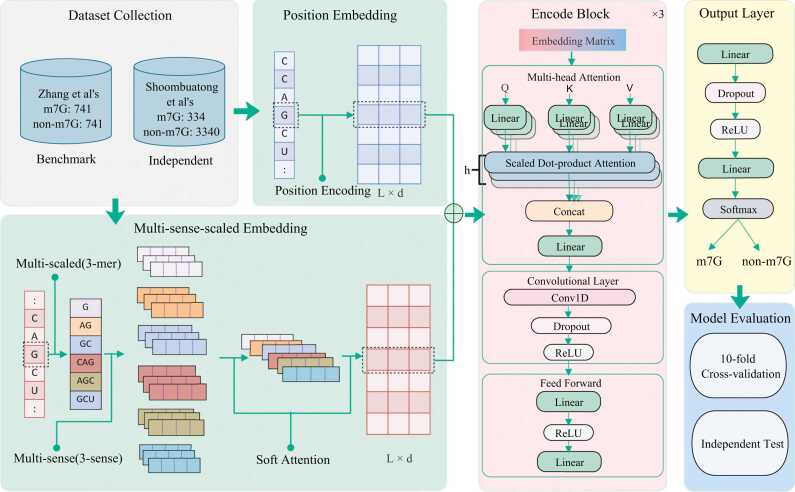
The architecture of TMSC-m7G. In which, *L* and *d* represent the sequence length and the dimensionality of the embedding features, respectively, while *h* denotes the number of heads in the multi-head attention mechanism.

In this investigation, we present a predictor built upon the transformer framework [35] that uses adaptive learning feature representation methods and deep learning algorithms, namely TMSC-m7G. Firstly, we use a multi-sense-scaled word embedding method, which is combined with the verified position embedding as the representation vector of sequence, to extract the context information and sequential information respectively. Then, we use the encoder in transformer for encoding. It is worth noting that we introduce a convolutional layer in the encode block to enhance the capture of local feature details. Refer to Fig. 1 for details on the model structure. The experimental findings indicate the effectiveness and feasibility of our model design. Through 10-fold cross-validation and independent test, TMSC-m7G has achieved better prediction results than existing state-of-the-art predictors.

## 2 Materials and methods

### 2.1 Datasets

Datasets play an important role in model training. In this study, we utilize the dataset introduced in the prior research [25] as the benchmark dataset for our model, which was discovered by Zhang et al. [36] through experimental studies in human HeLa and HepG2 cell lines, containing 801 RNA sequences with N7-methylguanosine modification sites. The optimal length of the sequence is 41 bp according to the previous works [27], [36], [37], [38] with the m7G position located at the center and flanked by 20 bp upstream and downstream. Sequences were deduplicated using the CD-HIT [39], [40] program with an 80% threshold, resulting in 741 positive samples. To obtain balanced data, a matching quantity of negative samples was chosen randomly to form the final dataset, which serves as our benchmark dataset for training and testing model. During the training process, the dataset is divided randomly into training and testing sets, maintaining an 8:2 ratio. Additionally, to facilitate a direct comparison with existing models, we conduct independent testing using the same imbalanced dataset of positive and negative samples as employed in the work [28]. The inclusion of a higher count of negative samples in the dataset also mimics real-world scenarios, providing a robust evaluation of the model. This dataset comprises m7G sequences from the HeLa cell line obtained from m6A-Atlas, with redundant sequences with similarity greater than 80% removed, resulting in 334 positive samples. An additional 3340 negative samples were constructed using the procedure described by Chen et al. [25]. For specifics about the dataset, consult (Table 1).

**Table 1:** Details of benchmark dataset and independent dataset.

Dataset	Sequence length	Number of m7G	Number of non-m7G
Benchmark dataset	41-bp	741	741
Independent dataset	41-bp	334	3340

### 2.2 Model overview

TMSC-m7G is constructed under the transformer framework [35], which consists of three components: embedding model, encode block and output layer. The embedding model is tasked with converting the sequence into a digital vector format for the machine to comprehend. In particular, we adopt the multi-sense-scaled word embedding method proposed by He et al. [41]. Compared with ordinary word embedding, it can better learn contextual information and different semantic information of sequences, which is helpful in enhancing the model's performance. In addition, since the order information of the words in the statement is usually missing in common token embedding, we consider position embedding. The output of the embedded module is obtained by adding the token embedding and position embedding vectors. The second part is the encode block. In transformer framework, the basic components of the encoder are multi-head attention mechanism and feedforward network composed of linear layers. Transformer has the advantage of parallel computing and a strong ability to capture global information, but it is weaker than Recurrent Neural Network (RNN) [42], [43] and Convolutional Neural Network (CNN) [44] in extracting local information. Therefore, we introduce a convolutional layer to the encode block to better capture the local information of sequences. The final part is the output layer, which consists of the linear layers, Dropout, ReLU, and Softmax. Please refer to Fig. 1 for a model diagram. A detailed description of each module is described below. Regarding the training of TMSC-m7G, you can refer to Algorithm 1.

### 2.3 Embedding model

#### 2.3.1 Multi-sense-scaled embedding

Multi-sense-scaled embedding is a word embedding technique, which is mainly used to map words into high-dimensional vector spaces to capture their semantic and syntactic information. Compared with traditional word embedding methods, multi-sense-scaled embedding can deal with multiple semantics of words and offer additional valuable information for the model.

To achieve multi-sense-scaled embedding, according to He et al.'s work [41], first of all, for multi-scale embedding, we need to convert the sequence into k-mer. It means that the bases in the sequence are divided into word combinations according to a sliding window of a certain size. For example, for the sequence *R*= ’*CAGCU*’, its 2-mer is.

(1) *R*_*2-mer*_ = [‘*CA*’, ’*AG*’, ’*GC*’, ’*CU*’]

Considering the running cost, we set k = 1, 2, 3 to obtain the corresponding embedding of the three scales 1-mer, 2-mer, and 3-mer, respectively. Three scales generate 1, 2, and 3 embedding vectors, respectively. Therefore, each base corresponds to six embeddings. In the next step, each embedding is represented as three embeddings with different senses. Subsequently, a soft attention mechanism is employed to fuse multiple embeddings. The soft attention mechanism allocates attention weights to different embeddings, balancing the importance of these embeddings [45], [46].

Multi-sense embedding assumes that various embeddings of the same base signify different senses of the base, and assigning multi-sense embeds to the base can enhance the representation ability of the feature. During random initialization, we assign multiple representation vectors to the same base and then use the soft attention mechanism for fusion. Similarly, we set the number of senses as 3. When the two strategies of multi-scale and multi-sense are combined, that is, multi-scale embedding is carried out separately on the three senses simultaneously, then the three dimensions are fused by soft attention to get a one-to-one vector for each base. To help understand, see Fig. 1.

Through multi-sense and multi-scale embedding technology, we can better capture the complex semantics and diversified usage of words, and improve the performance of natural language processing tasks.

#### 2.3.2 Position embedding

Position embedding comes from Vaswani et al.'s work [35]. Transformer can’t utilize the sequential order information, thus position embedding is necessary. There are two methods of position embedding: adaptive position embedding that can be learned, and fixed position encoding. Vaswani et al.'s tested the two methods in the work [35] and found that the outcomes from both methods exhibited similarity. In order to save the cost of running time, we choose the fixed position encoding, which means to derive the embedding vector by formula.

The position embedding vector (*PE*) for a sequence of length L can be calculated by the formula (2) as follows

(2) $$\{\begin{array}{c} PE_{\left(pos,2i\right)}=\;\sin\left(\frac{pos}{b^{2i/d_{\mathrm{model}}}}\right)\\ PE_{\left(pos,2i+1\right)}=\;\cos\left(\frac{pos}{b^{2i/d_{\mathrm{model}}}}\right) \end{array}$$

where *pos* is the position of base in the sequence (0≤pos≤L−1), and *i* ($0\leq {i<d}_{\mathrm{model}}/2$) represents each position of the *PE* vector. The *b* and d_model_ are constants, where d_model_ represents the dimension of the final embedding vector, and it should be equal to the dimension of token embedding. The b and d_model_ values are set to 1000 and 64 respectively in this study.

### 2.4 Encode block

Encode block is the core part of the model. Traditional transformer fully uses attention mechanism and has good parallel computing capability and global information acquisition capability, but lacks local information capturing. While CNN has good local information acquisition capability and complementary effect with attention mechanism. Therefore, we incorporate a convolutional layer between the multi-head attention mechanism and the feedforward network within the encode block. The compositions of the encode block are multi-head attention mechanism, convolutional layer, and feedforward network in turn. The structure of the encode block can be seen in Fig. 1.

#### 2.4.1 Multi-head attention

Multi-head attention is a commonly used attention mechanism in deep learning. The main idea is to learn multiple attention in different representation spaces to capture different levels and aspects of information, thus aiming to improve the model's overall performance. Specifically, firstly, the input of the multi-head attention mechanism is linearly transformed by three matrices to derive three distinct matrices: the query matrix (*Q*), the key matrix (*K*), and the value matrix (*V*). These three matrices have the same dimension of d_molel_. Then, for each head *h*, matrix multiplication will be carried out on *Q*, *K,* and *V* respectively to get the score matrix. Meanwhile, to avoid the product value of the matrix being too large or too small, which may lead to the disappearance or explosion of the gradient in the backpropagation, it is also necessary to scale the score matrix after calculation. Specifically, we divide the value of each element by the square root of the header dimension d_k_. This process is commonly known as Scaled Dot-product Attention [35]. The corresponding output matrix can be achieved by multiplication between the score matrix and the value matrix, which can be specifically expressed as the formula (3).

(3) $$Attention(Q,K,V)=soft\;\max\left(\frac{QK^{T}}{\sqrt{d_{k}}}\right)V$$

The softmax operates to convert scores into probability distributions, and generally $d_{k}=d_{\mathrm{mod}\;el}/h$.

The attention operation is carried out in each head, and the results of multiple heads are spliced at last. Finally, the final output is obtained through a linear transformation.

(4) $$head_{i}=Attention(QW_{i}^{Q},KW_{i}^{K},VW_{i}^{V})$$

(5) $$Multihead(Q,K,V)=Concat(head_{i},.,head_{h})W^{O}$$

where the $W_{i}^{Q}$,$W_{i}^{K}$,$W_{i}^{V}$represent the weight matrix of the linear transformation of the three matrices of the *i*-th header respectively, $W^{O}$is the weight matrix of the linear transformation of the output matrix.

#### 2.4.2 Convolutional layer

Convolutional layer serves as the backbone of CNN. Through convolution kernel, the convolutional layer performs convolutional operations on the input to extract the convolution results of different features of the input data, forming a new matrix, so as to realize the extraction of the original data features. In this work, the feature matrix output by the multi-head attention mechanism is input into the convolutional layer to further extract information. In order to mitigate the risk of overfitting in the model, a dropout layer is added and activated by the ReLU function. The specific operation is shown in the formula (6).

(6) Convolution(X)=ReLU(Dropout(WX+b))

#### 2.4.3 Feedforward network

The feedforward network is implemented in transformer encoder using a two-layer fully connected network. By mapping the input vector to a space of higher dimensions, the model can learn more complex nonlinear mapping relations, thus improving the model expression ability.

### 2.5 Output layer

After obtaining the final output of the encode blocks, the final feature extracted by the model is fed into the final output layer, which consists of two fully connected layers, and the predicted probability value is acquired through the softmax function.

### 2.6 Performance evaluation metrics

To evaluate the model's performance, we select several commonly used evaluation metrics: Accuracy (ACC), Precision (Pr), Sensitivity (Sn), Specificity (Sp), F1 score (F1), Area Under Curve (AUC), and Matthews Correlation Coefficient (MCC) [47], [48], [49], [50], [51]. ACC assesses the percentage of accurately categorized samples relative to the total sample count, and a higher ACC indicates improved classification effectiveness of the model. Pr measures how many of the samples predicted as positive by the model are indeed true positives. A value closer to 1 indicates that the model is more accurate when predicting samples as positive. By measuring the ratio of true positive samples correctly identified as positive by the classifier, Sn evaluates the strength of the classifier in recognizing positive samples, with a higher Sn indicating superior performance. Sp measures the percentage of actual negative samples correctly predicted as negative by the classifier, revealing the recognition ability of the classifier towards negative samples. A higher Sp indicates a stronger recognition capability of the classifier towards negative samples. F1, ranging from 0 to 1, signifies a superior equilibrium between Precision and Recall when closer to 1, indicating enhanced model performance. AUC refers to the area under ROC curve, and the horizontal and vertical coordinates of each point on ROC curve respectively represent the corresponding false positive rate and true positive rate under different thresholds. The larger the AUC, the better the classifier performance. MCC takes into account the classification effect of positive and negative samples and ranges from [− 1, 1]. MCC is not affected by uneven data set distribution and class proportion. As the value approaches 1, it indicates that the classifier has a better performance. Additionally, we evaluated the loss values (Loss) between predicted values and true values. Each metric is calculated by the formula (7).(7)where TP, TN, FP, and FN correspondingly denote true positives, true negatives, false positives, and false negatives.

## 3 Results and discussion

### 3.1 Experimental settings

The cross-entropy is used as a loss function to calculate the loss, and the weight parameters of TMSC-m7G are optimized by the Adam [52] algorithm. The batch size is 32, the learning rate is 0.0001, and the iteration epoch number is 100. In all dropout layers, the ratio is 0.5. As for the three important hyper-parameters: the number of encode blocks, the dimension of word embedding vector, and the number of heads of multi-head attention mechanism, we will select optimal values through experiments. See Table 2 for more details on the experimental parameter settings.

**Table 2:** Experimental parameter settings of TMSC-m7G in detail.

Structure		Parameter settings	Input shape	Output shape
Embedding model	Token embedding	Number of senses in multi-sense: 3 Number of k (-mer) in multi-scaled: 3 Embedding dimension: 64	(B,-)	(B,43,64)
	Position embedding	The constant b: 1000 The constant $d_{\mathrm{model}}$ (embedding dimension): 64	(B,-)	(B,43,64)
Encode block	Multi-head attention	Number of heads in multi-head attention: 8 Embedding dimension of vector k, q, v- 32	(B,43,64)	(B,43,64)
	Convolutional layer	Kernel number: 1 Kernel size: 1 Stride: 1 Padding: 0	(B,43,64)	(B,43,64)
	Feedforward network	Hidden layer dimension: 64	(B,43,64)	(B,43,64)
Output layer	Linear	Hidden layer dimension: 16	(B,64)	(B,2)
	Softmax	----	(B,2)	(B,2)

### 3.2 Parameter analysis

**Fig. 2:**
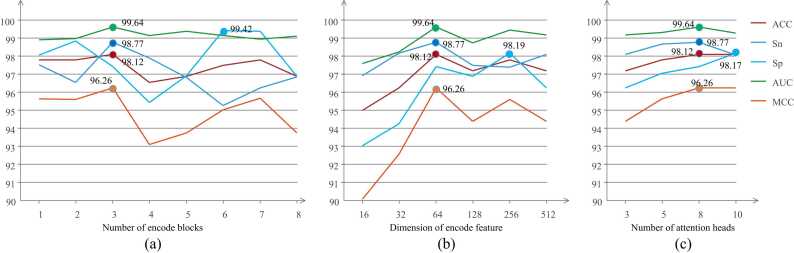
Experimental results of hyper-parameter setting. (a) Experiment on the number of encode blocks. (b) Experiment on the dimension of encode feature. (c) Experiment on the number of attention heads.

The setting of the hyper-parameters will impact the predictive performance of the model. In order to find the better combination of hyper-parameters that can make the prediction better, we conduct some experiments to analyze several important hyper-parameters. The first is the number of encode blocks, which is set as 1–8 to verify the performance of corresponding models respectively and evaluate with evaluation metrics. Based on the experimental results, the model's predictive performance doesn't exhibit improvement despite increasing the number of layers; it maintains a consistent level. According to the comprehensive experimental results, the model demonstrates better performance with 3 encode blocks. The second is the dimension of encoded feature, as well as the dimension of embedded feature. We have verified the dimensions as 16, 32, 64, 128, 256, and 512 respectively. According to the comprehensive results, when the dimension is 64, the prediction effect is the best. Finally, we also verify the influence of the number of heads of the multi-head attention mechanism on the model and obtain the evaluation metrics when the number of heads is 3, 5, 8, and 10 respectively. As per the results, the model exhibits optimal effectiveness with 8 heads. To sum up, we finally set the number of encode blocks, dimension of encode feature, and number of attention heads as 3, 64, and 8 respectively to ensure the performance of the model. The specific results of the parameter experiment are shown in Fig. 2.

### 3.3 Ablation study

**Fig. 3:**
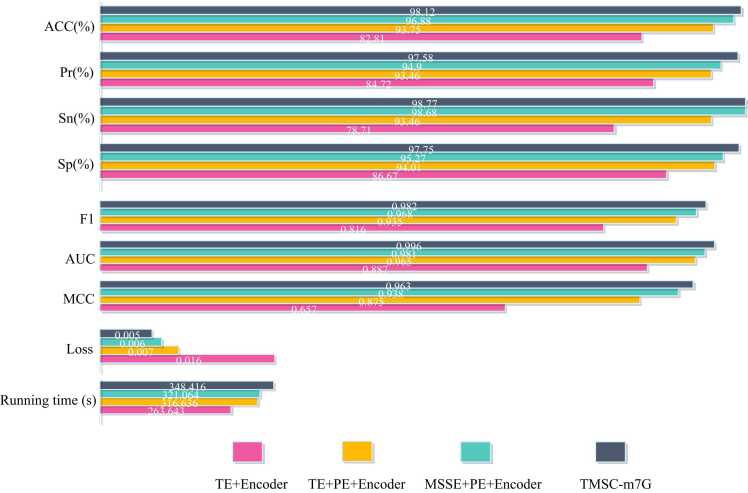
Comparison of evaluation metric values for ablation experiments on model structures.

**Fig. 4:**
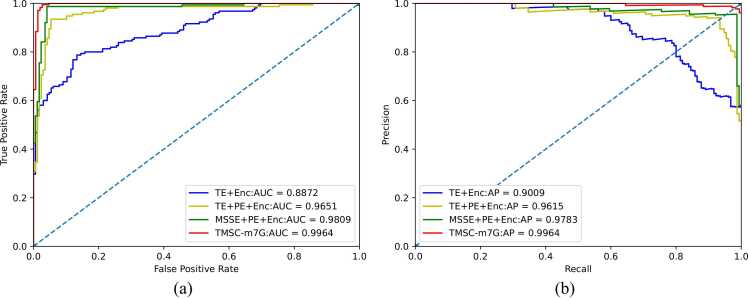
ROC and PR curves for ablation experiments on model structure. (a) ROC curves of models corresponding to different structures. (b) Precision-Recall curves of models corresponding to different structures.

To confirm the validity of the model structure of TMSC-m7G, we conduct ablation experiments on the model structure. We construct the structures with traditional token embedding and traditional encoder (TE + Encoder), traditional token embedding and position embedding and traditional encoder (TE + PE + Encoder), multi-sense-scaled embedding and position embedding and traditional encoder (MSSE + PE + Encoder), multi-sense-scaled embedding and position embedding and encoder with convolutional layer (TMSC-m7G). The comparative analyses among these four models are conducted to validate the crucial roles of position embedding, multi-sense scaled embedding, and convolutional layer in the complete model. Each model is separately trained and tested for this purpose. As the model's structure becomes more complex, the runtime of the model under the same operating environment is also increasing. However, the predictive metric values of the experimental results indicate that TMSC-m7G outperforms other model structures, demonstrating the effectiveness of our model. In addition, position embedding significantly impacts the model's performance, underscoring the relevance of sequential information. Fig. 3 and Fig. 4 display the details of the results obtained from the ablation experiments.

### 3.4 Performance comparison with different methods

#### 3.4.1 Performance on benchmark dataset

To gauge the predictive efficacy of TMSC-m7G, experiments are conducted using a 10-fold cross-validation method [53] on the benchmark dataset. The experimental findings are outlined in Table 3. The cross-validation results of TMSC-m7G are compared with those of other existing models in the same subject. Specifically, iRNA-m7G [25], XG-m7G [27], m7G-IFL [7], and THRONE [28] are used for comparison, which are mostly based on traditional sequence feature encoding methods and machine learning algorithms. To ensure a fair comparison, the experimental results of other models were directly taken from the original paper, which presumably reflects the authors' optimized parameter settings. The outcomes are detailed in Table 4, indicating that our model exceeds the performance of others in seven evaluation metrics, among which ACC, Pr, Sn, SP, F1, AUC, and MCC reach 98.70%, 98.60%, 98.85%, 98.58%, 0.9872, 0.9955 and 0.9740 respectively. Compared with THRONE, which is the state-of-the-art method, our ACC, Sn, SP, AUC, and MCC have respectively improved by 3.70%, 3.85%, 3.48%, 0.0295, and 0.0740. This shows that our model has better performance. TMSC-m7G learns more efficient adaptive representations from RNA sequences and achieves more accurate predictions than conventional features. In addition, deep learning algorithm performs better than machine learning method. Our preconception about the ability of encode blocks to capture characteristic information is validated.

**Table 3:** 10-fold cross-validation results on the benchmark dataset.

k	ACC (%)	Pr (%)	Sn (%)	Sp (%)	F1	AUC	MCC	Loss
1	98.75	98.81	98.81	98.68	0.9881	0.9962	0.9749	0.0031
2	98.75	98.77	98.77	98.73	0.9877	0.9987	0.9750	0.0023
3	100.0	100.0	100.0	100.0	1.000	1.000	1.000	0.0017
4	97.50	97.7	97.7	97.26	0.9770	0.9877	0.9496	0.0035
5	99.38	100.0	98.78	100.0	0.9939	0.9984	0.9876	0.0030
6	95.63	93.42	97.26	94.25	0.9530	0.9836	0.9128	0.0052
7	98.55	97.33	100.0	96.92	0.9865	0.9956	0.9713	0.0026
8	100.0	100.0	100.0	100.0	1.000	1.000	1.000	0.0015
9	100.0	100.0	100.0	100.0	1.000	1.000	1.000	0.0028
10	98.44	100.0	97.14	100.0	0.9855	0.9943	0.9690	0.0035
Mean	98.70 ± 0.7960	98.60 ± 1.226	98.85 ± 0.6812	98.58 ± 1.132	0.9872 ± 0.008349	0.9955 ± 0.003302	0.9740 ± 0.01591	0.0029 ± 0.0006

**Table 4:** Performance comparison with state-of-the-art methods on benchmark dataset by 10-fold cross-validation.

Methods	ACC (%)	Pr (%)	Sn (%)	Sp (%)	F1	AUC	MCC	Loss
iRNA-m7G	89.81	NA	88.66	90.96	NA	0.9460	0.8000	NA
XG-m7G	91.22	NA	91.48	90.96	NA	0.9720	0.8250	NA
m7G-IFL	92.50	NA	92.40	92.60	NA	0.9510	0.8500	NA
THRONE	95.00	NA	95.00	95.10	NA	0.9660	0.9000	NA
TMSC-m7G	98.70 ± 0.7960	98.60 ± 1.226	98.85 ± 0.6812	98.58 ± 1.132	0.9872 ± 0.008349	0.9955 ± 0.003302	0.9740 ± 0.01591	0.0029 ± 0.0006

#### 3.4.2 Performance on independent dataset

To validate the generalization ability and practicability of the TMSC-m7G, we also conduct independent dataset tests. The model trained with the training dataset is saved, and then the prediction results are obtained directly without any training on another set of independent data. The results of the data in Table 5 show that the prediction accuracy of our model reaches 92.92% on the independent dataset, which has excellent generalization ability. Furthermore, compared to the current leading methods, our model improves on most of the metrics. This basically shows that our model showcases superiority against other models.

**Table 5:** Performance comparison with state-of-the-art methods on independent dataset.

Methods	ACC (%)	Pr (%)	Sn (%)	Sp (%)	F1	AUC (%)	MCC	Loss
iRNA-m7G	84.90	NA	61.40	87.20	NA	NA	0.371	NA
XG-m7G	80.90	NA	57.20	83.30	NA	80.20	0.2880	NA
m7G-IFL	32.40	NA	36.80	32.00	NA	32.90	− 0.1880	NA
THRONE	88.60	NA	87.70	88.70	NA	87.10	0.5680	NA
TMSC-m7G	92.92	61.14	60.77	96.14	0.6096	91.87	0.5707	0.0084

### 3.5 Visualization of learned features

**Fig. 5:**
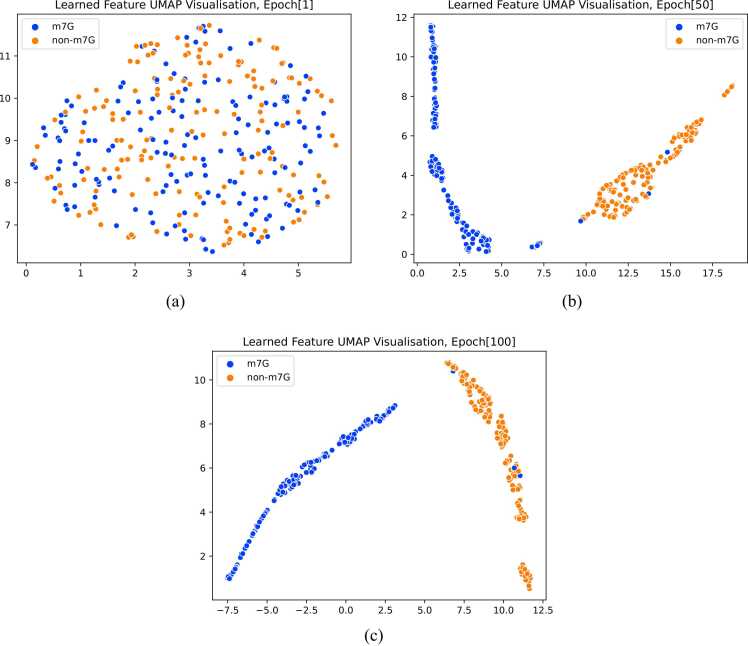
UMAP visualization of features learned by TMSC-m7G. (a) Features learned by the model when epoch is 1. (b) Features learned by the model when epoch is 50. (c) Features learned by the model when epoch is 100.

To ensure the model's interpretability and verify the validity of the features extracted from the model, we use the Uniform manifold approximation and projection (UMAP) tool [54] to visualize the acquired features from TMSC-m7G. UMAP is a nonlinear dimension reduction algorithm that maps high-dimensional data to low-dimensional space and preserves nonlinear structure and topology information between data. We visualize the data points on a two-dimensional plane, where different colors represent the data points with different labels. Fig. 5 displays the features learned by the model after the first epoch, 50th epoch, and 100th epoch, given a total number of epochs set to 100. Observing Fig. 5, it's evident that the distribution of sample points is chaotic at the initial input of the model. In the middle of training, the samples are gradually clear and distinguishable, and in the final iteration, they are roughly separated according to labels. This shows that our model learns effective features that help with classification.

### 3.6 Sequence analysis

**Fig. 6:**
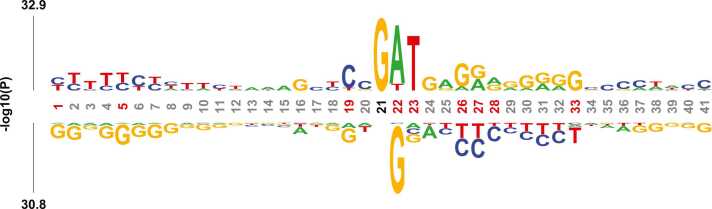
Probability Logo diagram of positive sample sequences obtained by kpLogo visualization of sequence.

**Fig. 7:**
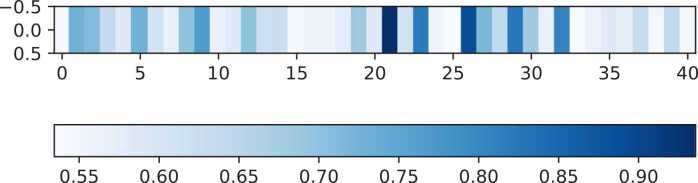
The importance of different positions in the sequence as determined by TMSC-m7G.

Finally, sequence analysis is performed to verify the interpretability of TMSC-m7G in the biological sense. Firstly, the kpLogo tool [55] is used to generate the Probability Logo diagram of RNA sequences, which is a commonly used graphical display tool for sequence comparison. It can help users better understand the similarities and differences between sequences by presenting the information of various sequence motifs at different locations, and thus better understanding their biological implications. Probability Logo can be used to find significant sites or fixed sites related to specific biological functions in biological sequences. We generate the Probability Logo of positive sample sequences, as shown in Fig. 6. The positions of red coordinates in Fig. 6 represent significant positions, which often have a significant impact on the structure and function of sequences. In order to verify the validity of TMSC-m7G from this perspective, the predicted probability of each position output in the model is taken as an element in the matrix, and then distinct colors represent various ranges of values, and heat map is generated to show the regions that are paid more attention by the model. Specifically, the darker the color, the higher the predicted value of the position, that is, the higher the model pays attention to it, and vice versa. Illustrated in Fig. 7, we generate the corresponding heat map of the data predicted as positive samples. By comparing with the Probability Logo, the region that the model allocates more attention to roughly matches the significant region of the sequence, which indicates that our recognition model is correct and effective. From the perspective of biological significance, TMSC-m7G is reliable and feasible.

## 4 Web-server

**Fig. 8:**
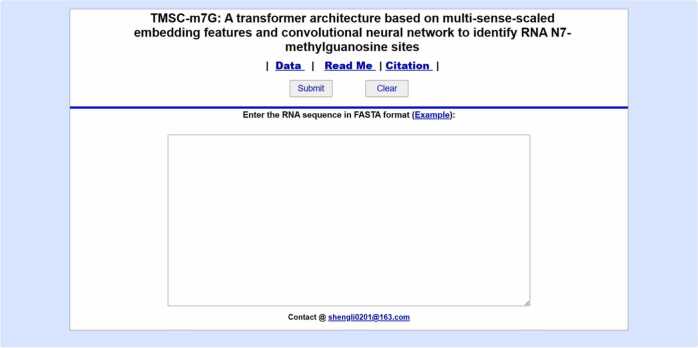
The web-server prediction interface for TMSC-m7G.

To make TMSC-m7G convenient for more users and improve the practicability of TMSC-m7G, we develop a user-friendly online server. Biologists can use web server to get the results of m7G sites prediction without any complex mathematical calculations. The web page is shown in Fig. 8. In this procedure, users simply input RNA sequences and click “Submit” to obtain the desired outcomes. It's important to note that the text box requires the input format to be FASTA; otherwise, an error will be generated. Users can view the standard RNA sequences input format by clicking on “Example”. If you want to re-forecast, click “Clear” to clear the prediction content and input the sequences to be predicted again. In addition, the “Data” option stores the datasets used in this study. “Read Me” and “Citation” options describe the usage guideline and attribution statement, respectively. The web server is available at http://39.105.212.81/. This server is open and free to all.

## 5 Discussion

We have proposed a novel tool for m7G sites prediction. However, in independent test experiments, we observed relatively lower values for certain metrics. Precision, sensitivity, and F1 score precisely evaluate the classifier's ability to identify positive samples. The observed weakness in recognizing positive samples is mainly attributed to the significantly lower number of positive samples compared to negative samples, leading to substantial errors in the classifier's identification of positive samples. Additionally, these metrics are influenced not only by sample imbalance but also by factors such as data distribution and classifier performance. The underlying root cause of this issue requires further in-depth investigation in future work.

The m7G prediction computational model holds extensive application prospects, contributing to in-depth investigations into the biological functions of RNA modifications, gene expression regulation, identification of disease biomarkers, advancement in drug development, support for personalized medicine, cancer research, and providing a robust tool for epigenetics, genomics, and transcriptomics. This offers potential opportunities to deepen our understanding of the mechanisms underlying RNA modifications and to develop novel therapeutic strategies.

## 6 Conclusions

The prediction of RNA N7-methylguanosine sites is instrumental for advancing the exploration of RNA's biological functions, unraveling the mechanisms of RNA modification, and understanding the interplay between RNA modification and diseases. This endeavor not only introduces novel avenues for research but also suggests potential directions for treating diseases through RNA modification. Therefore, a deep learning model based on transformer and natural language processing is introduced in this study to enhance the performance when predicting m7G sites. Firstly, we use multi-sense-scaled word embedding combined with fixed position embedding to carry out vector transformation of sequences. In this way, adaptive representation features outperform traditional ones, and in terms of extracting potential information of sequence context, it is better than ordinary word embedding methods. Next, we input the embedded vectors into an improved encode block consisting of multi-head attention mechanisms, convolutional layers, and feedforward networks to better capture global and local information of sequences. Finally, the prediction results are obtained from the linear layers. The experimental results demonstrate the superiority of our model over the current state-of-the-art models. In the independent dataset, the performance is also better than other models, the ACC value increased by 4.32%. In addition, we conduct ablation experiments to verify the rationality of the structural design of the model and select several important hyper-parameters through parameter experiments. Finally, we carry out some visual analysis to illustrate the model’s interpretability. From the aforementioned studies, we predict that TMSC-m7G will be a useful tool for m7G site prediction, which will facilitate the development of RNA functional site identification. However, there is still room for improvement. We are committed to further research to obtain a higher-quality model.

Algorithm 1 TMSC-m7G training.

**Table:** 

## CRediT authorship contribution statement

**Shengli Zhang**: Methodology, Software, Investigation, Supervision, Writing – review & editing. **Yujie Xu**: Data curation, Writing – original draft, Visualization. **Yunyun Liang**: Software, Validation, Writing – review & editing.

## Declaration of interests

The authors declare that they have no known competing financial interests or personal relationships that could have appeared to influence the work reported in this paper.
